# Control of the proportion of inner cells by asymmetric divisions and the ensuing resilience of cloned rabbit embryos

**DOI:** 10.1242/dev.152041

**Published:** 2018-04-18

**Authors:** Dimitri Fabrèges, Nathalie Daniel, Véronique Duranthon, Nadine Peyriéras

**Affiliations:** 1 BioEmergences Laboratory, CNRS USR 3695, 91190 Gif-sur-Yvette, France; 2 UMR BDR, INRA, ENVA, Université Paris Saclay, 78350, Jouy en Josas, France

**Keywords:** Rabbit pre-implantation development, Somatic cell nuclear transfer, Digital specimens, Spatial cell segregation, Asymmetrical divisions, Cell death, *In silico* experimentation, 3D+time 2-photon imaging

## Abstract

Mammalian embryo cloning by nuclear transfer has a low success rate. This is hypothesized to correlate with a high variability of early developmental steps that segregate outer cells, which are fated to extra-embryonic tissues, from inner cells, which give rise to the embryo proper. Exploring the cell lineage of wild-type embryos and clones, imaged *in toto* until hatching, highlights the respective contributions of cell proliferation, death and asymmetric divisions to phenotypic variability. Preferential cell death of inner cells in clones, probably pertaining to the epigenetic plasticity of the transferred nucleus, is identified as a major difference with effects on the proportion of inner cell. In wild type and clones, similar patterns of outer cell asymmetric divisions are shown to be essential to the robust proportion of inner cells observed in wild type. Asymmetric inner cell division, which is not described in mice, is identified as a regulator of the proportion of inner cells and likely gives rise to resilient clones.

## Abstract (precis)

**Summary:** A unique quantitative approach based on complete reconstruction of the cell lineage that unveils an unknown mechanism of size control in cell populations of rabbit blastocysts, wild types or clones.

## INTRODUCTION

Variability coincides with the possibility of adapting to changing environments (Darwin, 1859). Consistently, wild-type populations are intrinsically variable (Raj and van Oudenaarden, 2008). The production of inbred strains, as achieved in laboratory conditions in mice, aims to minimize genotypic and phenotypic variability (Beck et al., 2000). Animal cloning by somatic cell nuclear transfer (SCNT) has been developed to go a step further, by keeping desired traits and producing clones in different mammalian species (Hochedlinger and Jaenisch, 2002; Inoue et al., 2005; Wakayama et al., 1999). However, the cloning efficiency is low (Hochedlinger and Jaenisch, 2003; Yang et al., 2007) and much effort has been devoted to improving its success rate. Following SCNT, embryonic development eventually resumes and leads to a normal organism. However, whether the developmental path of clones falls within the normal range of embryonic variability, in terms of cell identity, proliferation, division orientation and death, remains to be explored.

Quantitative studies investigating multiscale phenotypic variability in bacteria (Elowitz et al., 2002; So et al., 2011; Taniguchi et al., 2010), yeasts (Blake et al., 2003; Carey et al., 2013) and metazoans (Boettiger and Levine, 2009; Ohnishi et al., 2014; Wernet et al., 2006) have been published previously. However, the quantification of variability at the level of genetic expression and cell behavior in mammalian embryos relies mainly on the observation of fixed specimens. The current challenge is to achieve the *in vivo* and *in toto* multiscale observation of developing embryos, in order to perform a systematic quantitative analysis of phenotypic traits and model the multiscale variability. The cellular scale of organization is expected to integrate variation at the subcellular level (e.g. thermal agitation and stochastic gene expression) as well as cues from the macroscopic organization (e.g. mechanical constraints) and from environmental conditions. Long-term *in toto* imaging of pre-implantation mammalian embryos has been recently reported in mice (Strnad et al., 2015), with a difficult trade-off between photodamage (Squirrell et al., 1999) and achieving the spatial and temporal resolution required to produce the full automated reconstruction of cell lineage and cell shapes as is possible in other species (Amat et al., 2014; Faure et al., 2016; Fernandez et al., 2010). Mammalian embryos develop from fertilization to the blastocyst stage in a few days, segregating two cell populations distinguished by their position and presumptive fate. Outer cells form an epithelial layer that is fated to form extra-embryonic tissues. Inner cells form a cluster in the blastocoel cavity that gives rise to the embryo proper. Although the same organization is observed in almost all mammalian species, possible differences in underlying cell behaviors is largely unknown. Additionally, the possibility to extrapolate our knowledge to humans requires investigating biological diversity. In this context, the rabbit *Oryctolagus cuniculus* has been described as more similar to human than the mouse, for certain phenotypic traits (Duranthon et al., 2012; Okamoto et al., 2011; Piliszek et al., 2017).

We have investigated the variability of cell dynamics in normal and cloned rabbit embryos from the entire cell lineage reconstructed from two-photon microscopy images throughout pre-implantation stages. The quantitative comparison of cell death, cell proliferation and division orientation in inner and outer cell populations highlights defects and possible resilience in clones. The asymmetric division of inner cells, which has not yet been described in the mouse, is shown to have the appropriate patterns to regulate the size of the inner cell population observed at the time of hatching. This putative mechanism would not, however, be able to compensate for the most severe inner cell death cases. The epigenetic state of donor cells and their ability to give rise to embryonic cells adapted to the cellular environment of both the inner and outer domains of the developing blastula is likely to be at stake.

## RESULTS

**Fig. 1.**
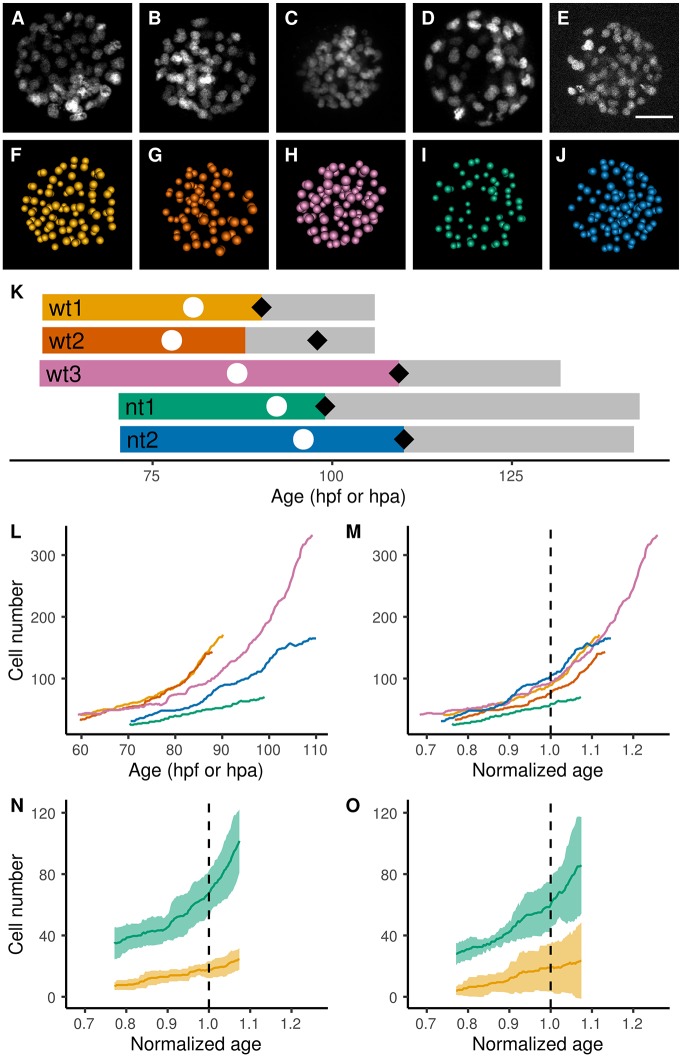
**Reconstruction of digital rabbit specimens from 3D+time imaging of labeled nuclei.** (A-M) wt1 (yellow), wt2 (orange), wt3 (red-purple), nt1 (blue-green) and nt2 (blue). (A-E) *Z*-projection of 3D volumes, 15 min before the first blastocoel collapse. (F-J) *Z*-projection of reconstructed embryos at the same timestep. Each approximate nucleus center is represented by a sphere. Scale bar: 50 µm. (K,L) Age based on fertilization time (hpf, wild-type embryos) and activation time (hpa, clones). (K) Time line of the imaging sequences. The cell lineage validation and curation is limited to the colored part. White disc, first collapse; black diamond, hatching. (L) Cell number over time without temporal rescaling. (M-O) Cell number over time with temporal rescaling based on the fertilization or activation time and first blastocoel collapse. Dashed line, first blastocoel collapse. (N) Average number of inner cells (yellow line) and outer cells (blue-green line) in wild-type embryos. Standard deviation is shown as the paler area. (O) Average cell number in clones (inner cells, yellow; outer cells, blue-green).

Digital specimens were obtained from 3D+time imaging of three wild-type embryos (wt1-3) and two clones (nt1,2) that were nuclear stained by injection at the one-cell stage of synthetic mRNA encoding H2B-EGFP and developing from the 32-cell stage until hatching (Fig. 1, Movies 1 and 2). RNA concentration and imaging conditions were optimized and did not affect embryo survival and cell growth (Table S1 and Fig. S1). Clones were obtained by SCNT using cumulus cells taken from the oocyte donor female. Fully curated cell lineages were submitted to an automated analysis of spatiotemporal characteristic features (Fig. 1F-J, see Materials and Methods).

The cell number over time (Fig. 1L) showed different paces from one embryo to another, impairing further inter-individual comparison. We therefore normalized the embryo developmental speed. Temporal rescaling based on morphogenetic events led to the best fit in terms of cell number and embryonic volume evolution (Fig. S2). Fertilization (for wild type) or activation (for clones) and the first blastocoel collapse were taken as fixed points, 0 and 1, respectively, for the temporal rescaling of the different specimens and the calculation of a normalized age (n.a.) (Fig. 1M, Table S2 and Movie 3). The comparison between the different specimens is thereafter limited to the common period from 0.768 to 1.073 n.a. Cell growth in clones appeared slower than that of wild type (12% and 17% slower for nt1 and nt2, respectively). In addition to growing slower than the wild types, the two clones differed from each other, whereas the three wild-type embryos looked very similar in terms of growth rate. However, as viable clones have been shown to form kits of normal size (Challah-Jacques et al., 2003), we assumed that differences in growth rate could be compensated for at later stages and we expected to find more-severe abnormalities to explain the very low success rate of rabbit cloning.

Because variations in single-cell positions from one embryo to another prevented the identification of individual cells across specimens, the inner and outer cell populations appeared to be the relevant level of organization to use to further compare embryonic cell dynamics. Although the average inner and outer cell numbers were similar between wild type and clones, the latter had a higher inner cell number standard deviation (Fig. 1N,O). Despite the small number of embryos supporting this observation, we hypothesized that the variability of the inner cell number may correlate with the low survival rate described in SCNT embryos, as an insufficient number of inner cells leading to a smaller inner-to-total cell ratio would compromise embryonic development, as described in mice (Morris et al., 2012).

**Fig. 2.**
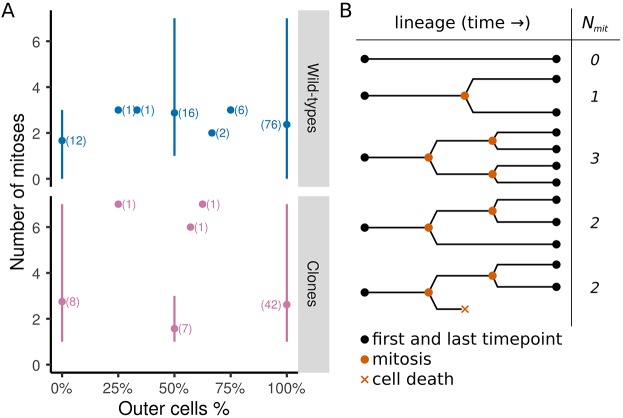
**Number of mitoses and cell identity along the cell lineage.** (A) Average number of mitoses as a function of the identity of a cell in each cell clone: each cell at the onset of the observation window gives rise to a cell clone with a proportion of inner and outer cells, depending on mitosis type and cell death. The percentage of cells of each type is plotted against the corresponding number of mitoses (solid lines to indicate the minimum and maximum number of mitoses and dots to indicate the average number of mitoses). (Top) Wild-type embryos (*n*=114 cells). (Bottom) Clones (*n*=60 cells). (B) Typical binary trees are schematized with different patterns of cell division and death (temporal progression from left to right). *N_mit_*: number of mitoses in the cell clone.

**Fig. 3.**
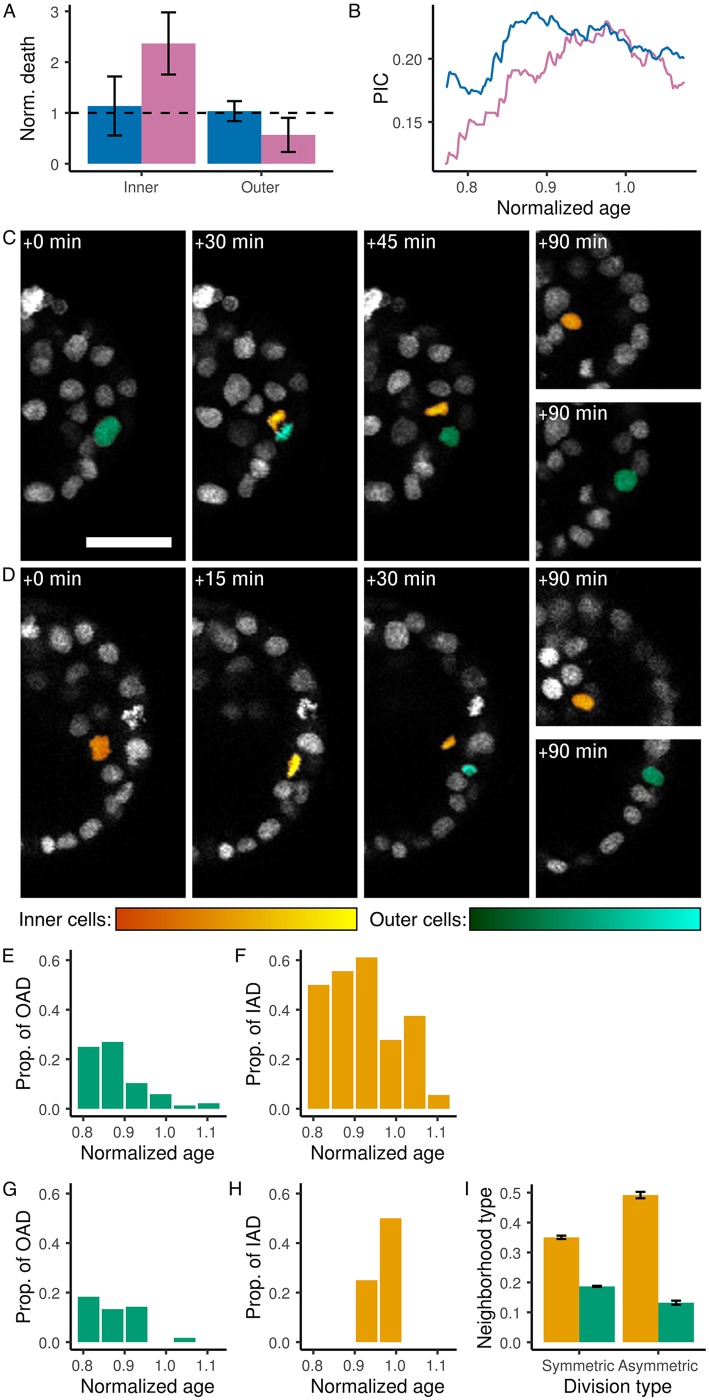
**Contribution of cell death and asymmetric divisions to the proportion of inner cells.** (A) Normalized death ratio (Norm. death) for inner and outer cells in wild-type embryos (blue) and clones (red-purple). Standard error is indicated by the black solid line. Dashed line indicates the normalized death ratio if cell death and cell type are not correlated. (B,E-I) Time in n.a. (B) Average proportion of inner cells (PIC) in wild type (blue) and clones (red-purple). (C) Example of an outer cell asymmetric division in wt3 (by 91.75 hpf≈1.058 n.a.). (D) Example of an inner cell asymmetric division in wt3 (by 101.5 hpf≈1.171 n.a.). Scale bar: 50 µm. (E-H) Bin size 0.05 n.a. ≈4.3 h. Outer cells in blue-green. Inner cells in yellow. (E,G) Proportion of asymmetric divisions in outer cells (OAD). (F,H) Proportion of asymmetric divisions in inner cells (IAD). (E,F) Wild-type embryos. (G,H) Clones. (I) Average proportion of cells of the other type in close proximity to dividing cells whether symmetrical (left plots) or asymmetrical (right plots). Standard error is indicated by the black solid line.

Looking for an explanation of the variability of the inner cell number appeared to be relevant for further comparison of wild type and clones. This variability was not explained by differences in their cell division rate (Fig. 2 and Fig. S3), but we noted a highly variable number of inner cells at the onset of the observation (Fig. S4), indicating that the overall inner cell number would thereafter be highly sensitive to cell death and/or asymmetric divisions. A division is identified as asymmetric when daughter cells are found in different compartments (inner and outer) (see Materials and Methods). Cell death was clearly identified in 3D+time imaging data (Movie 4). It was confirmed using the TUNEL assay that the proportion of cell death in inner or outer cell populations was similar in injected, non-injected and imaged embryos (Fig. S1). Cell death obviously had dramatic consequences on the size of already small populations. We observed this type of incidence for nt1 and wt3 during the first 0.1 n.a. (30% and 39% inner cell death for populations of three and nine cells, respectively: Fig. S4G,I,J,L). Overall, we did not observe any characteristic temporal distribution pattern of cell death to discriminate between wild-type embryos and clones. The spatial distribution of cell death, however, indicated a marked bias for inner cell death in clones, with a normalized death ratio of 2.32 (s.d.=0.03) in inner cells compared with 0.63 (s.d.=0.26) in outer cells, whereas cell death was independent of cell type in wild-type embryos (normalized death ratio of 1.03 for inner cells with s.d.=0.90 and 1.06 for outer cells with s.d.=0.31) (Fig. 3A). Inner cell death bias thus appeared as a major difference between clones and wild-type embryos. Surprisingly, this bias did not prevent clones from achieving a proportion of inner cells similar to that of wild types (Fig. 3B). Asymmetric cell division was the only possible process remaining to explain how the proportion of inner cells, which was initially much lower on average in clones compared with wild-type embryos, increased then plateaued before the first collapse (Fig. 3C-H, Fig. S5 and Table S3) at a value close to the average observed in wild types and possibly corresponding to a critical threshold (Morris et al., 2012). Asymmetric divisions were identified for both outer cells and inner cells (Movies 5 and 6). The distribution of asymmetric divisions in outer cells feeding the inner cell population was very similar in clones and wild type. Consequently, this could not explain the increase in the proportion of inner cells observed in clones, rising from half of the value observed in wild type at the onset of the observation sequence to the same value by 0.93 n.a. The best explanation of the recovery of the clones seemed to come from the distribution of the asymmetric divisions of inner cells. Although asymmetric divisions of inner cells were observed in both wild type and clones, very few were observed in the latter, and only at late stages. Asymmetric divisions of inner cells, not yet described in mice, were found preferentially for cells close to the outer layer (Fig. 3I), suggesting positional and or mechanical cues. We propose that the asymmetric division of inner cells functions as an ultimate regulation process to balance the proportion of inner cells in both wild type and clones.

**Fig. 4.**
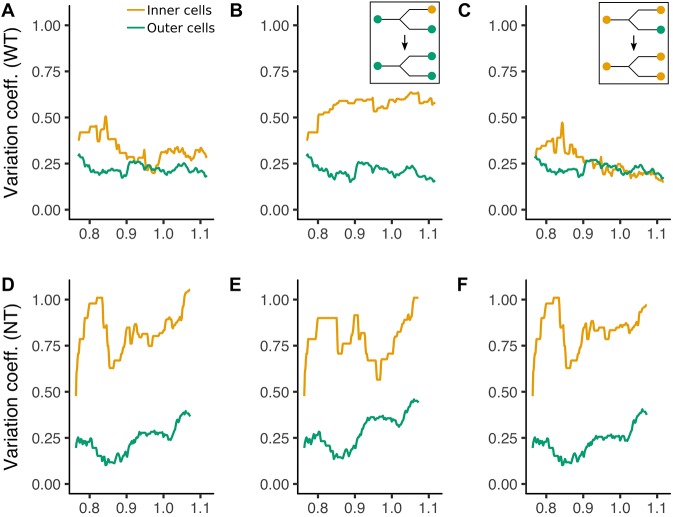
**Impact of the transformation of asymmetric divisions into symmetric divisions on the variability of inner and outer cell populations.** (A-F) Variation coefficient as a function of time in n.a. in wild type (A-C) and in clones (D-F) for inner cells (yellow) and outer cells (blue-green). (A,D) Normal conditions. (B,E) Outer cell asymmetric divisions transformed into symmetric divisions as shown in the inset. (C,F) Inner cell asymmetric divisions transformed into symmetric divisions as shown in the inset.

The putative contribution of the asymmetric divisions of inner cells to regulating the size of the inner cell population is supported by the transformation of asymmetric divisions into symmetric ones *in silico* and the simulation of the corresponding cell lineages (Fig. 4 and Fig. S6). By the onset of the imaging sequence, the inner cell population has already been built by the earlier asymmetric divisions of outer cells, so that the simulation does not impair the evolution of cell population that relies on symmetric divisions. The variability of the outer cell population, estimated by the standard deviation over the mean cell number, remains low in both wild types and clones in experimental conditions. The variability remains low upon simulation, with the transformation of asymmetric divisions into symmetric divisions either for outer cells (Fig. 4B,E) or for inner cells (Fig. 4C,F). Thus, the size of the outer cell population is robust regarding the contribution of asymmetric divisions. Conversely, the size of the inner cell population in wild types becomes early on (0.85 n.a.) highly variable upon transformation of the observed asymmetric of outer cells divisions into symmetric divisions. We interpret that, in the latter case, the simulation reveals the intrinsic variability of the inner cell number in relation to extent of cell death. This variability is consistent with the inner cell population variability observed in clones both in experimental conditions and upon simulation. We hypothesize that the bulk of outer cell asymmetric divisions build the inner cell population and, depending on the extent of cell death, the asymmetric divisions of inner cells adjust the size of the inner cell population and the inner to total cell ratio. Consistently, the evolution of the inner to total cell ratio in simulated lineages (Fig. S6), similar in wild types and clones upon transformation of the asymmetric divisions of outer cells, fits with the delayed and scarce asymmetric divisions of inner cells in clones. Our interpretation is that the normal regulation of the inner cell population size leads to a trade-off between robust embryonic development and the space of variability that is potentially available for resilience.

## DISCUSSION

The cell lineage phenomenology of rabbit pre-implantation embryogenesis lays the basis for comparison with and extrapolation to human. Similar studies in other species are, however, yet to be produced. Available live-imaging data in mice only encompasses the first six divisions (Strnad et al., 2015). Our data focused on later stages starting by the fifth generation. Rabbit development was impaired when imaged earlier, possibly correlating with the timing of zygotic activation. Our study was limited by embryo survival after mRNA injection and long-term imaging (3 days). Nuclear transfer further compromised the success rate. In addition, only 20% of embryos imaged until hatching produced an exploitable full cell lineage, owing to various imaging artefacts.

Comparing individuals first required a temporal rescaling. Taking the first collapse as a landmark led to similar patterns of asymmetric divisions in outer cells, validating our choice and also indicating some internal constraints in the relative timing of specific morphogenetic events. Our time-lapse imaging data suggest a wave of asymmetric divisions in rabbit embryos between the 32- and 64-cell stages. The presence of inner cells at the onset of our imaging sequences indicates that earlier waves may have happened. In the mouse, at least two waves have been described by the 8- to 16-cell stage and the 16- to 32-cell stage (Johnson, 1981; Johnson and McConnell, 2004; Morris et al., 2012; Sutherland et al., 1990; Wennekamp and Hiiragi, 2012). The occurrence of a third wave by the 32- to 64-cell stage has been mentioned (Morris et al., 2010). In any case, this last wave of asymmetric division of outer cells is essential for forming the inner cell mass in a robust manner, as highlighted by our *in silico* transformation of the asymmetric divisions of outer cells into symmetric divisions. A more-striking difference between the mouse and the rabbit may lie in the occurrence of asymmetric inner cell divisions. We hypothesize that the ratio of asymmetric inner cell divisions is regulated to reach and maintain an optimal value of the proportion of inner cells. The hypothesis of such a regulatory scenario is supported by the observation of clones where an initial low proportion of inner cells correlates with a low ratio of asymmetric inner cell divisions. In this context and given the importance of this ratio for proper development, asymmetric inner cell divisions should also be found in the mouse.

The low survival rate observed in SNCT rabbit embryos should be consistent with major differences between viable and non-viable specimens in quantitative parameters, including cell proliferation and cell death. Our observations suggest that inner cell death is the major issue. The higher cell death rate observed in the inner cell population of clones could lead to a higher variability of their inner cell population compared with wild types that is sufficient to explain their low survival rate (Hochedlinger and Jaenisch, 2003; Yang et al., 2007). These observations relate to the epigenetic state of the transferred nucleus and the transfer protocol that brings the membrane and cytoplasm of the donor cell to the egg. The epigenetic state of the transferred nucleus may confer limited potency to the blastomeres, leading to a better survival rate in the trophectoderm than in the inner cell mass. The adaptive capacity of the donor nucleus to fit both the inside and outside environments is probably the major issue. It should be noted that IPS cells or ES cells that might be expected to solve this issue are not currently available in the rabbit. More generally, animal cloning by somatic cell nuclear transfer is a powerful strategy for assessing cell plasticity and how cell-environment interactions can affect cell selection.

## MATERIALS AND METHODS

### Animals and embryo culture

Experiments were performed in accordance with the International Guiding Principles for Biomedical Research involving animals, as promulgated by the Society for the Study of Reproduction and the European Convention on Animal Experimentation. Researchers working with the animals possessed a license delivered by the French veterinary services.

New Zealand White female rabbits (*Oryctolagus cuniculus*) were superovulated as described by Henrion et al. (1997) and mated with normal males (giving rise to wild-type embryos) or vasectomized males (for clones). One-cell stage embryos were flushed from oviducts with PBS at 19 h postcoitum (hpc). Synthetic mRNA at a concentration of 75 ng/µl in water was injected at the one cell stage at 38.5°C in M199 medium with 10% FBS, 0.5% penicillin/streptomycin and 20 mM HEPES (bM199-FBS). Embryos were kept at 38.5°C under 5% CO_2_ in bM199-FBS without HEPES (M199-FCS) with Phenol Red in a humid incubator until the morula stage. Embryos were then transported for imaging in a drop of bM199-FBS in a capillary. The temperature was maintained at 38°C during the transportation to the imaging location, where embryos were kept in M199-FBS.

### Assessing cell death

Chromatin fragmentation and cell behavior, including abnormal nucleus displacement, interrupted mitosis and disruption of the nuclear envelope, were used to annotate cell death in 3D+time imaging data. A TUNEL assay (12156792910, Sigma-Aldrich) was used to assess cell death in embryos cultured until 69 hpc, 73 hpc and 93 hpc, and fixed with phosphate-buffered saline (PBS; 0.1 M at pH 7.5; 189112-04, Invitrogen) and 2.5% paraformaldehyde for 1 h at 4°C. Embryos were rinsed in 1× PBS and permeabilized in 1× PBS, 0.5% Triton X-100 for 1 h at room temperature. After 1 h incubation in the TUNEL reaction mix at 38°C, embryos were rinsed in 2× SSC, then 1× PBS. Nucleus counterstaining was achieved using 4 µM Hoechst-33342 (H1399, Invitrogen) in PBS for 15 min. Embryos were rinsed in PBS and imaged immediately using a Zeiss confocal LSM-780. Hoechst-33342 and TMR-Red were excited at 405 nm and 543 nm. TMR-red signal was used to annotate apoptotic cells.

### Cloning by nuclear transfer

Rabbit oocytes without cumulus cells were enucleated as described previously (Challah-Jacques et al., 2003). Cumulus cells were introduced into the subzonal space of enucleated oocytes. Cell-oocyte pairs were electrostimulated to induce fusion. One hour later, fused embryos were activated by a second set of electric pulses followed by 1 h of incubation with 5 µg/ml cycloheximide and 2 mM 6-dimethylaminopyridine in M199-FBS. Clones were then cultured as carried out for wild-type embryos.

### Embryo mounting and imaging

Imaging was performed with Leica DM5000 and DM6000 upright microscopes SP5 MLSM, equipped with an Olympus 20/0.95NA W dipping lens objective or a Leica 20/1NA W dipping lens objective. For wt1 and wt2, the field size was 455.68×455.68 µm in *x* and *y*, and 174.24 µm in *z*, with a voxel size of 0.89×0.89×1.76 µm. For wt3 and nt1, the field size was 529.41×529.41 µm in *x* and *y*, and 241.27 µm in *z*, with a voxel size of 1.034×1.034×1.049 µm. For nt2, the field size was 412.67°412.67 µm in *x* and *y*, and 222.36 µm in *z*, with a voxel size of 0.806×0.806×0.797 µm. Volumes were acquired every 15 min. Two-photon excitation at 980 nm was performed with a pulsed laser beam (Ti-Sapphire femtosecond oscillator Mai Tai HP, Newport Spectra-Physics). Laser power was automatically modulated with a motorized half-wave plate, a polarized cube and real-time feedback to maintain a relatively constant signal-to-noise ratio. Emission signal was filtered with a 680 nm short-pass filter (Semrock) to remove infrared reflection and a 525/50 nm band-pass filter (Semrock). Photon detection was carried out with hybrid detectors (Hamamatsu Photonics K.K., Japan; Leica, Germany). An OKO-lab H101 system was used for temperature control. An Okolab DGT-CO2 system was used to control CO_2_ concentration. Raw data movies were made with Fiji (Schindelin et al., 2012). Raw data are available online at bioemergences.eu/fabreges-et-al/.

### Digital reconstruction

Reconstructions were performed on single specimens. A manual region of interest (ROI) was made to enclose each specimen. Signal intensity outside the ROI was set to zero to mask other specimens in the field of view. Embryos were reconstructed with the BioEmergences Workflow as described previously (Faure et al., 2016). Nuclear centers were detected with the Difference of Gaussians algorithm, parameters were manually selected every 25 timesteps to best fit the raw data and interpolated over the whole imaging sequence. Tracking was carried out using the Simulated Annealing algorithm. Cell lineages were manually curated with the software Mov-IT (Faure et al., 2016) either until hatching (wt1, wt3, nt1 and nt2) or earlier when the fluorescent signal was too weak (wt2). Cells were annotated as inner cells or outer cells based on the nucleus position at the last time step. Annotations were then propagated backwards along the cell lineage and mitoses were checked for their type (symmetric or asymmetric). Raw and reconstructed data are available online at bioemergences.eu/fabreges-et-al/.

### Temporal rescaling

The time of fertilization of wild-type embryos was estimated as 8 h postcoitum, as measured previously (Pincus and Enzmann, 1935). The activation time for clones was set at the time of the second electric pulses. Fertilization and activation times were used to define the first fixed point for rescaling (time 0.0 n.a.). The overall embryo volume was estimated with the surface enveloping the outermost nuclei (convex hull), calculated using the R geometry package (CRAN.R-project.org/package=geometry). The first decrease of more than 10% in the hull volume from one time-step to the next was taken as the first blastocoel collapse (80.7 hpf, 77.7 hpf, 86.8 hpf, 92.3 hpa and 96.0 hpa for wt1, wt2, wt3, nt1 and nt2, respectively) and used as the second fixed point for rescaling (time 1.0 n.a.).

### *In silico* experiment

Considering the cell identity only, asymmetric divisions were transformed into symmetric divisions *in silico*. The cell lineages were modified making the identity of their daughters match the identity of their mothers. This operation was performed for all inner cells or for all outer cells, depending on the condition. Cell position or trajectory was not modified and only the resulting cell identity was compared between conditions.

### Analysis of the reconstructed data

CSV files containing information for cell position, temporal links and annotations (validation, inner, outer) were constructed and analyzed with custom R scripts. Annotation of cell divisions (symmetry or asymmetry) was determined based on the identity of mother and daughter cells prior to their next mitosis. If daughter cells did not divide by the end of the imaging sequence, their identity at the last time step was used. When measurements were analyzed from pools of embryos (wild type versus clones), they were resampled over time to obtain the same number of time points. The finest temporal resolution was preserved and data were interpolated linearly if necessary.

### R scripts

Custom R scripts were written to perform specific analyses with Base libraries (Team, 2015) and the geometry package (CRAN.R-project.org/package=geometry). Graphs and figures were generated using ggplot2 (Wickham, 2009) and rgl (CRAN.R-project.org/package=rgl) packages for R.

## Supplementary Material

Supplementary information

Supplementary information
