# Supplementary material for: Control of the proportion of inner cells by asymmetric divisions and the ensuing resilience of cloned rabbit embryos
Source: Development. 2018 Apr 18;145(8):dev152041. doi: 10.1242/dev.152041 (PMC5964649; doi:10.1242/dev.152041)
Supplement: Supplementary information [file develop-145-152041-s1.pdf]

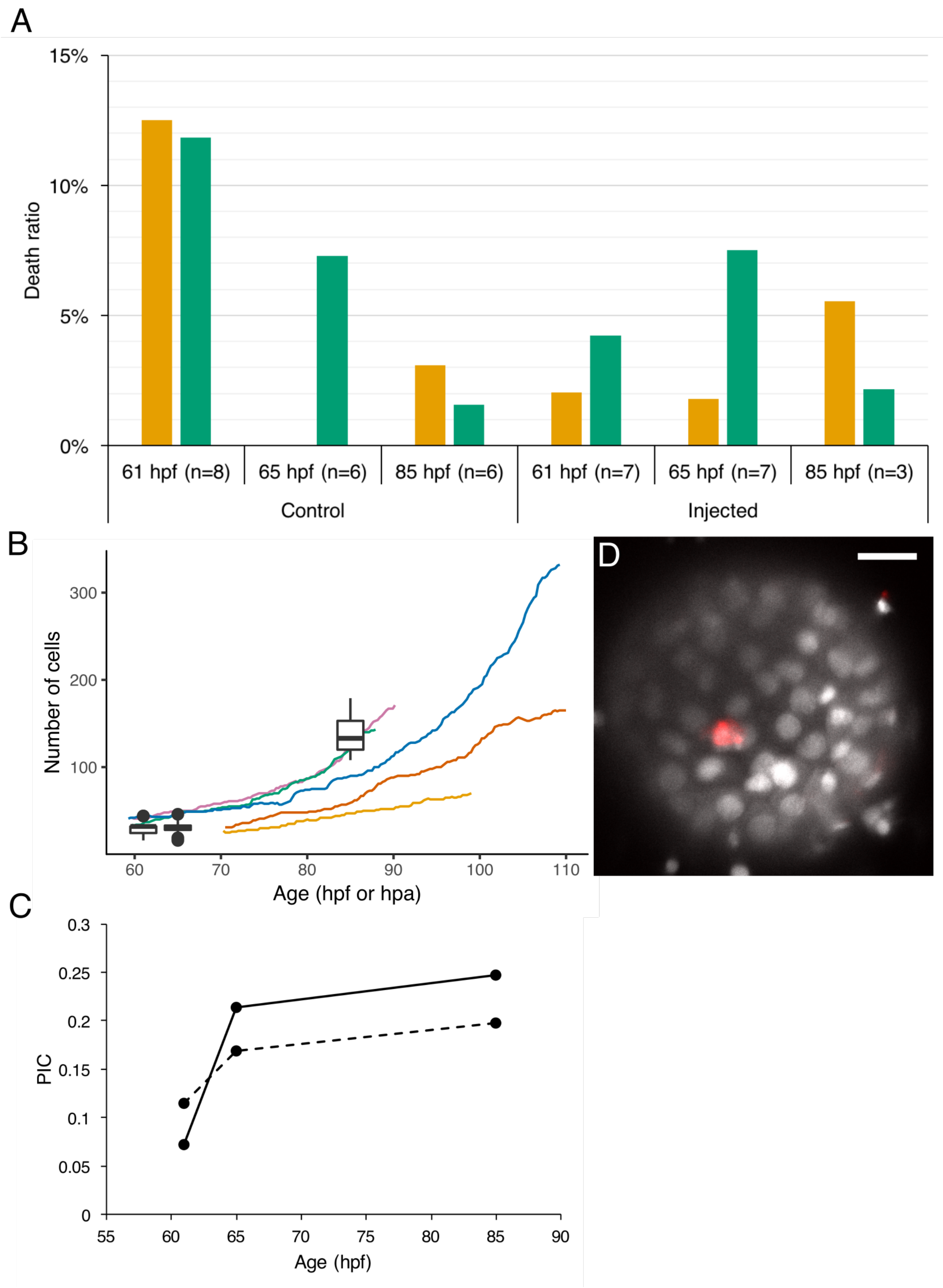

Fig. S1. Impact of RNA injection and 3D+time imaging on cell death and cell growth. (**A**) Proportion of TUNEL positive cells at 61 hpf, 65 hpf and 85 hpf in control and RNA-injected embryos, for inner cell (yellow) and outer cell populations (blue-green). (**B**) Total cell number in embryos fixed at 61 hpf, 65 hpf and 85 hpf (n-values as in **A**) to be compared with the growth curves of embryos wt1-3 and nt1-2 (color code as in Fig. 1) shown without temporal rescaling. (**C**) Proportion of inner cells (PIC) in wild-type embryos fixed at 61 hpf, 65 hpf and 85 hpf (n-values as in **A**). Solid line: non-injected embryos. Dashed line: H2B-EGFP mRNA injected embryos. The difference in the PIC between injected and non-injected embryos is not significant (Mann-Whitney U-test, p>0.05). (**D**) Sum Z projection 10 µm thick from a fixed non-injected embryo (85 hpf) with stained nuclei (Hoechst in gray) and TUNEL positive cells (in red). Scale bar: 25 µm.

Development • Supplementary information

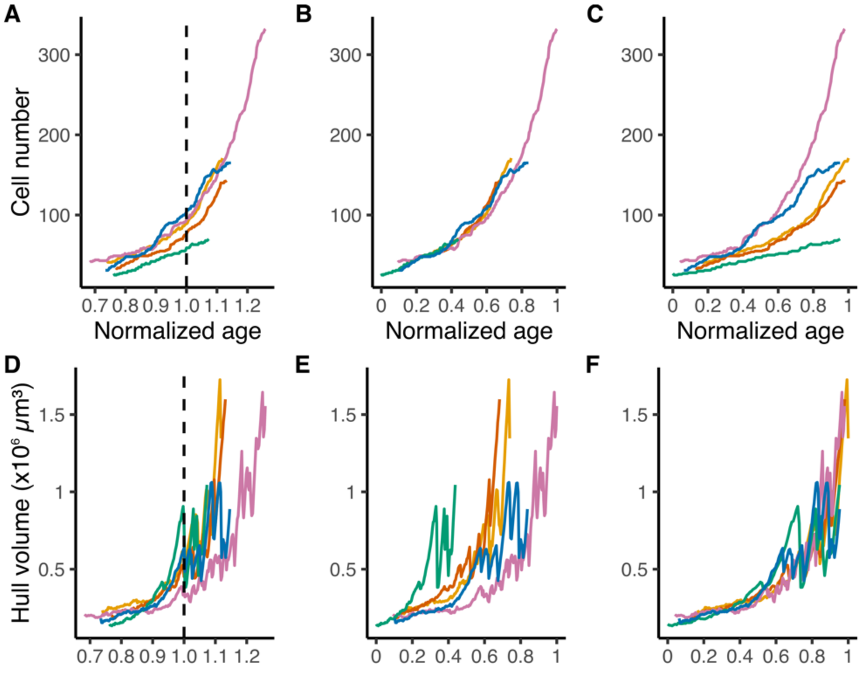

Fig. S2. Temporal rescaling. (**A**-**C**) Cell number over time. (**D**-**F**) Volume of the external envelope containing all the detected nuclei (embryo convex hull) over time. (**A**,**D**) Rescaling method based on morphogenetic events, i.e., fertilization or activation and first collapse (method 3 below). (**B**,**E**) Rescaling method based on the best exponential fit of cell number (method 1 below). (**C**,**F**) Rescaling method based on the best exponential fit of convex hull volume (method 2 below). Color code as in Fig. 1. To determine the best method, we calculated a distance between the curves for each method and for each pair of embryos. The distance was calculated as the average of the Euclidean distance between the two curves. The distance was normalized between 0% (smallest distance, best alignment) and 100% (largest distance, worst alignment) and an average score was calculated for volume and cell number alignment. Method 1 obtained a mean score of 32% (6% for cell number alignment and 57% for volume alignment). Method 2 obtained a mean score of 34% (53% for cell number alignment and 15% for volume alignment). Method 3 obtained a mean score of 28% (26% for cell number alignment and 30% for volume alignment) and was therefore used for further embryonic comparisons.

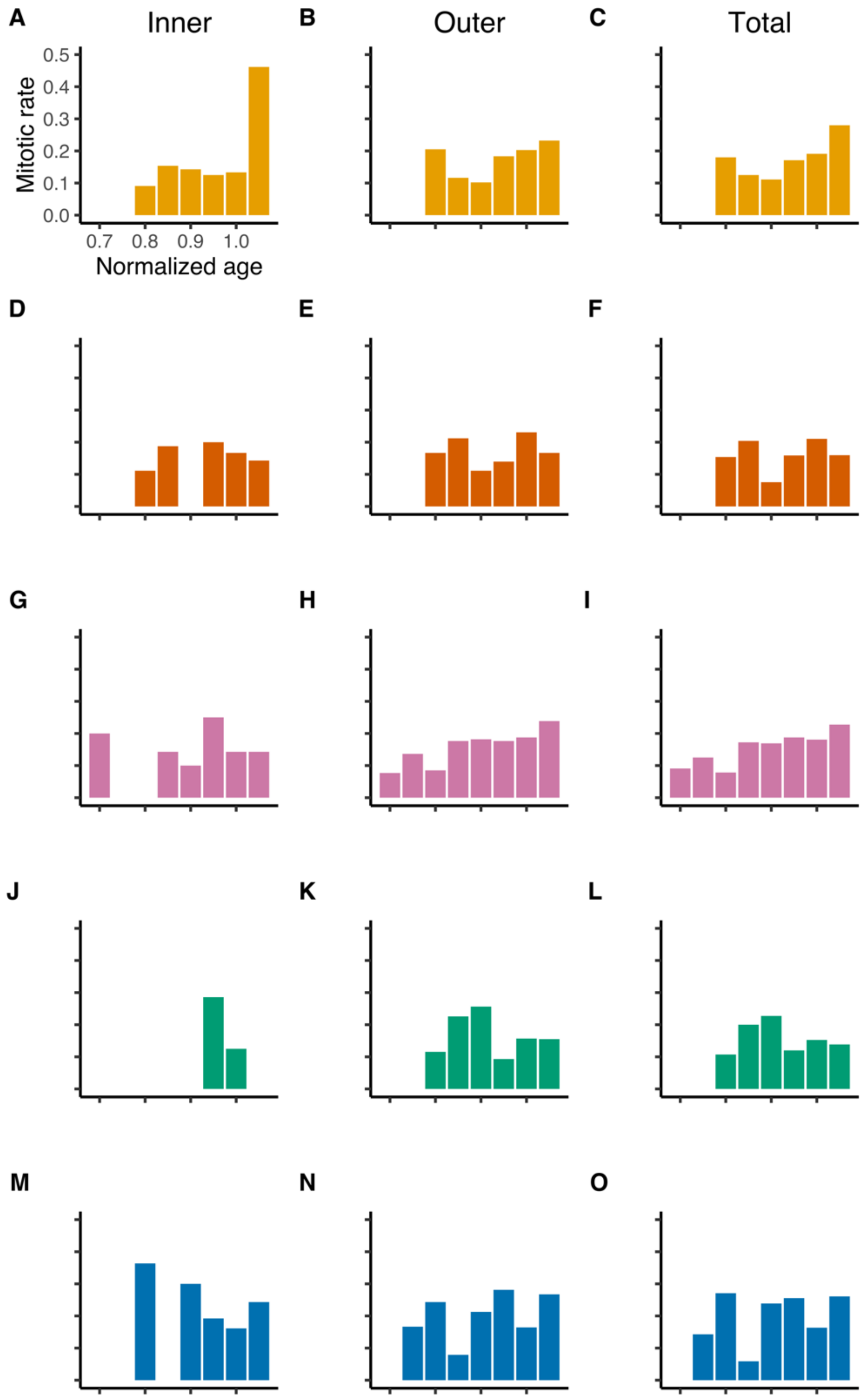

Fig. S3. Cell proliferation rate in wild-type embryos and clones over time (in n.a.). Profiles displayed for the five embryos wt1-3 and nt1,2 corresponding to rows 1 to 5 respectively. Color code as in Fig. 1. Bin size 0.05 n.a. ≈ 4.3 hours. (**First column**) Inner cell population. (**Second column**) Outer cell population. (**Third column**) All cells.

Development • Supplementary information

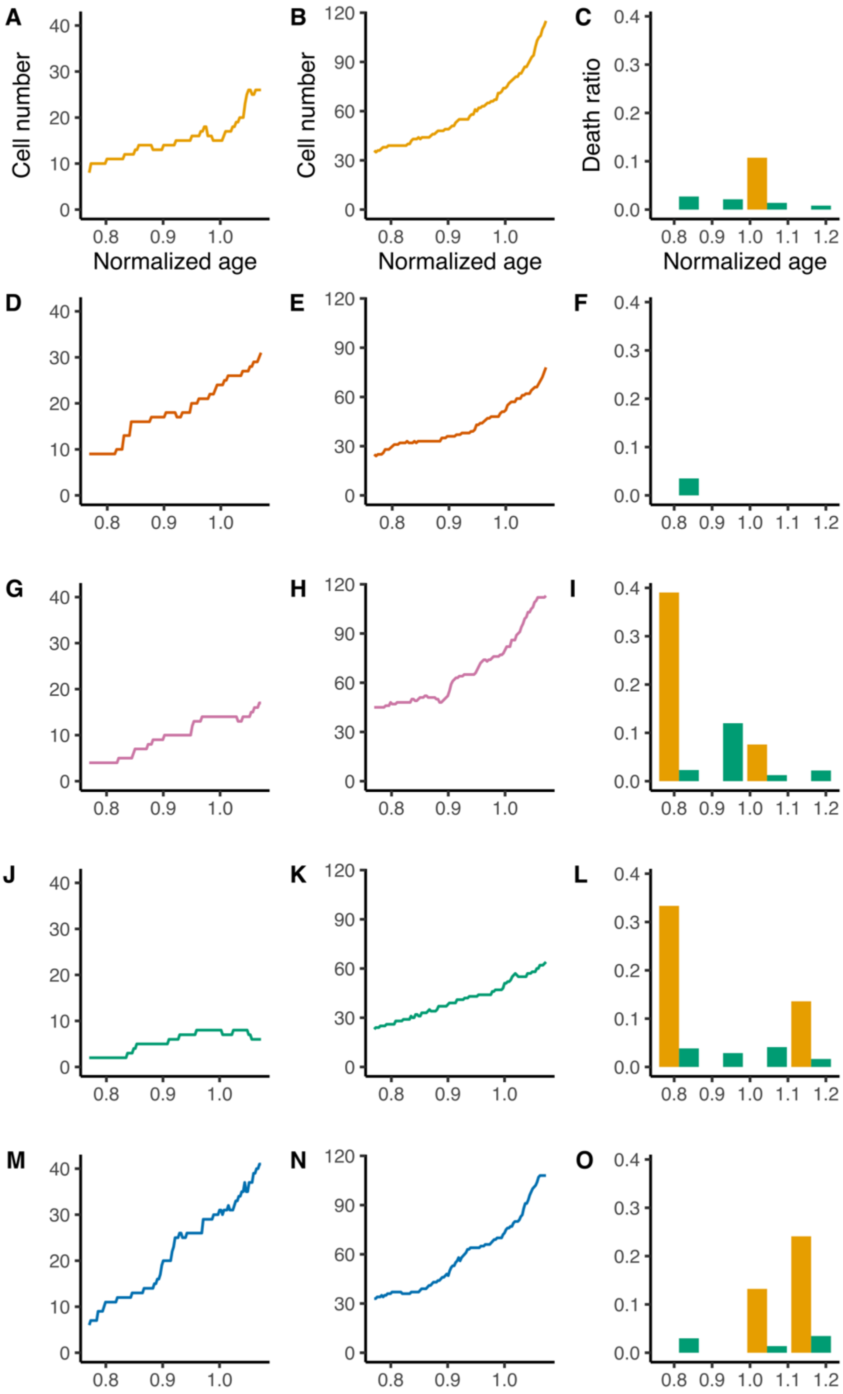

Fig. S4. Cell number and cell death rate along the cell lineage as a function of time (in n.a.). Profiles displayed for the five embryos wt1-3 and nt1,2 corresponding to rows 1 to 5 respectively. Color code as in Fig. 1. (**First column**) Inner cell number over time. (**Second column**) Outer cell number over time. (**Third column**) Cell death rate of inner cells (yellow) and outer cells (blue-green) over time, expressed as the proportion of cells dying during a given time period (bin size 0.1 n.a. ≈ 8.6 hours).

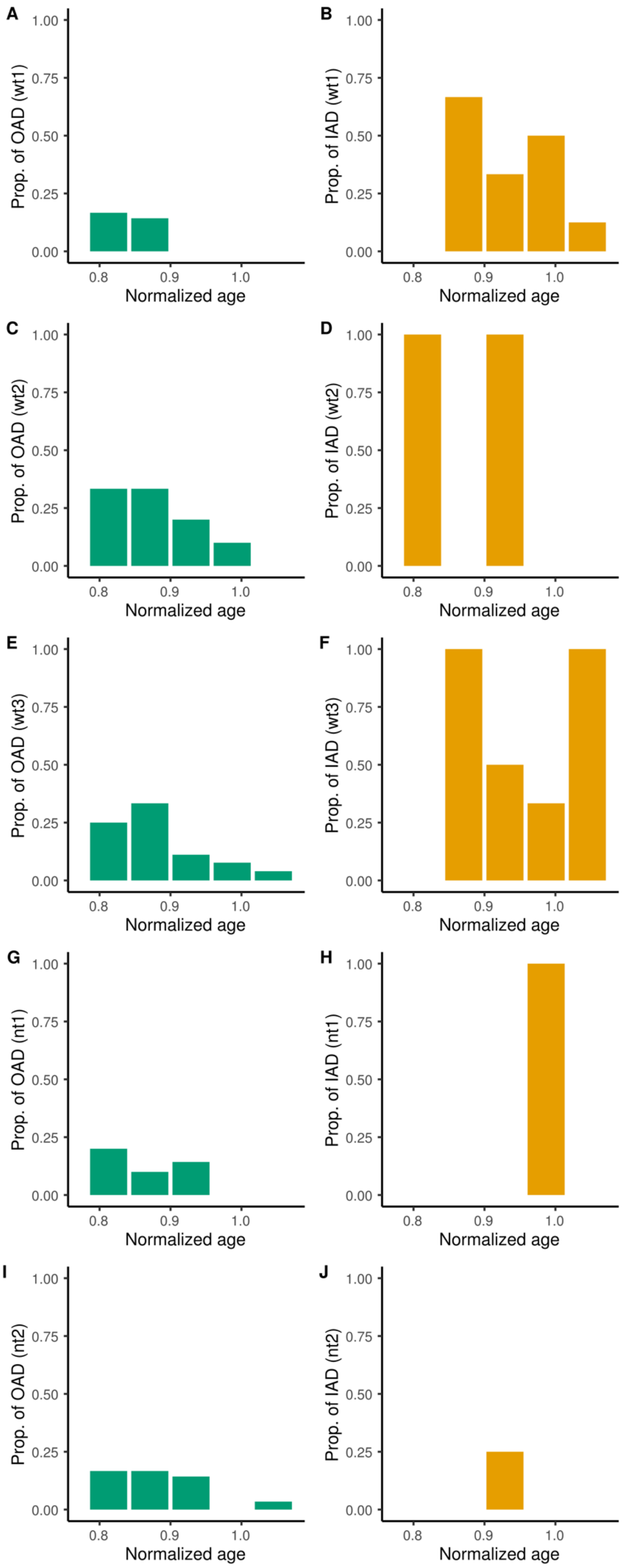

Fig. S5. Proportion of asymmetric divisions along the cell lineage as a function of time (in n.a.). Profiles displayed for the five embryos wt1-3 and nt1,2 corresponding to rows 1 to 5 respectively. Bin size 0.05 n.a. ≈ 4.3 hours. (**First column**) Proportion of outer asymmetric divisions (OAD). (**Second column**) Proportion of inner asymmetric divisions (IAD).

Development • Supplementary information

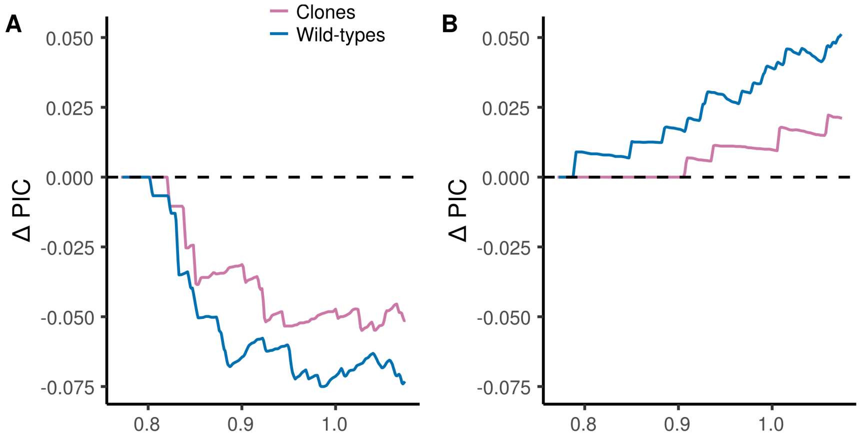

Fig. S6. Impact of the transformation of asymmetric divisions into symmetric ones on the proportion of inner cells over time (in n.a.). (**A-B**) Difference in the proportion of inner cells in WT (blue) and clones (reddish purple) as a function of time (in n.a.), dashed line for null difference. (**A**) Outer cell asymmetric divisions transformed into symmetric ones. (**B**) Inner cell asymmetric divisions transformed into symmetric ones.

Development • Supplementary information

**RNA concentration**	**10 ng/µL each**	**25 ng/µL each**	**75 ng/µL each**
**Non-injected**	96% (n=25)		
**H2B-mCherry + EGFP-RAS**	83% (n=6)	71% (n=7)	25% (n=8)
**H2B-EGFP**	-	-	85% (n=20)

Table S1. Survival rate of blastocysts either non-injected, or upon injection of different RNA combinations and concentrations.

Development • Supplementary information

**n.a.[1] (a.u.)**	**wt & nt[2] (h.p.f/a.)**	**wt[3] (h.p.f.)**	**wt1 (h.p.f.)**	**wt2 (h.p.f.)**	**wt3 (h.p.f.)**	**nt[4] (h.p.a.)**	**nt1 (h.p.a.)**	**nt2 (h.p.a.)**
0.65	**56h20**	**53h05**	52h25	50h30	56h25	**61h10**	59h60	62h25
0.70	**60h40**	**57h10**	56h30	54h20	60h45	**65h55**	64h35	67h10
0.75	**65h00**	**61h15**	60h30	58h15	65h05	**70h35**	69h10	71h60
0.80	**69h20**	**65h20**	64h30	62h10	69h25	**75h20**	73h50	76h45
0.85	**73h40**	**69h25**	68h35	66h00	73h45	**80h00**	78h25	81h35
0.90	**78h00**	**73h30**	72h35	69h55	78h05	**84h45**	83h00	86h25
0.95	**82h20**	**77h35**	76h40	73h45	82h25	**89h25**	87h40	91h10
1.00	**86h40**	**81h40**	80h40	77h40	86h45	**94h10**	92h15	95h60
1.05	**91h00**	**85h45**	84h40	81h35	91h05	**98h50**	96h55	100h45
1.10	**95h20**	**89h50**	88h45	85h25	95h25	**103h30**	101h30	105h35
1.15	**99h40**	**93h55**	92h45	89h20	99h45	**108h15**	106h05	110h25
1.20	**104h00**	**98h00**	96h50	93h10	104h05	**112h55**	110h45	115h10
1.25	**108h20**	**102h05**	100h50	97h05	108h25	**117h40**	115h20	119h60
1.30	**112h40**	**106h15**	104h50	100h60	112h50	**122h20**	119h55	124h45

Table S2. Correspondence between experimental age and normalized age (n.a.).

[1] Normalized age expressed in arbitrary units, with 0 as the fertilization or activation time, and 1 as the first blastocoel collapse.
[2] Average experimental age of both WT and clones.
[3] Average experimental age of WT embryos.
[4] Average experimental age of clones.

Normalized age	WT (3 embryos)				Clones (2 embryos)			
	Inner cells		Outer cells		Inner cells		Outer cells	
	Sym.	Asym.	Sym.	Asym.	Sym.	Asym.	Sym.	Asym.
**0.75 – 0.8**	1	1	9	4	3	0	8	1
**0.8 – 0.85**	3	3	15	6	0	0	8	2
**0.85 – 0.9**	3	0	17	2	4	2	16	4
**0.9 – 0.95**	5	4	27	2	6	1	21	1
**0.95 – 1.0**	6	2	41	1	5	1	20	0
**1.0 – 1.05**	13	5	59	1	9	0	35	1
**1.05 – 1.1**	6	2	36	1	5	0	13	0
**Total**	**37**	**17**	**204**	**17**	**32**	**4**	**121**	**9**
**Average per embryo**	**12.3**	**5.7**	**68**	**5.7**	**16**	**2**	**60.5**	**4.5**

Table S3. Number of symmetrical (Sym.) and asymmetrical (Asym.) divisions observed between 0.75 and 1.10 normalized age in inner and outer cells, in wild-type embryos and clones.

Development • Supplementary information

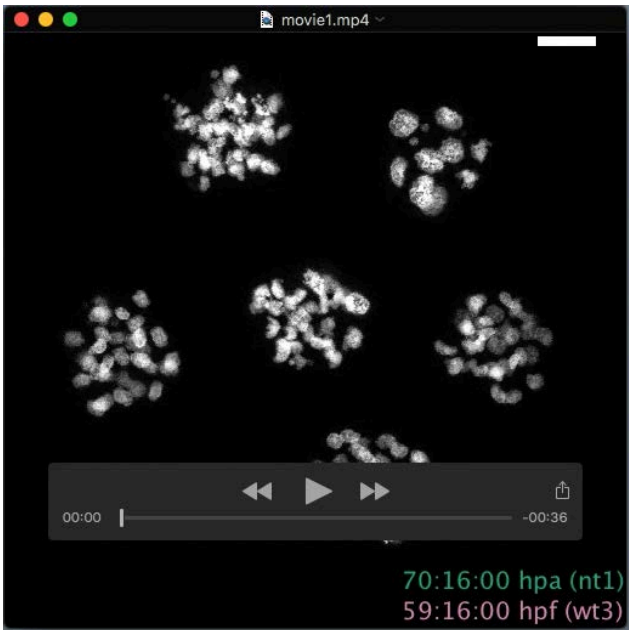

Movie 1. Imaging conditions. Development of six H2B-EGFP labeled embryos (4 wild types and 2 clones) over 50 hours under the microscope. The embryo in the middle is nt1. The top left embryo is wt3. The top right embryo is another clone, already dead before the onset of image acquisition. The three other embryos have a low signal-to-noise ratio (SNR) especially in inner cells and were not suitable for performing automated nucleus detection and tracking. The movie shows the hatching of nt1 (101.25 h.p.a.) through a small opening in its zona pellucida. The movie stops just before the hatching of wt3. Scale bar: 25 µm.

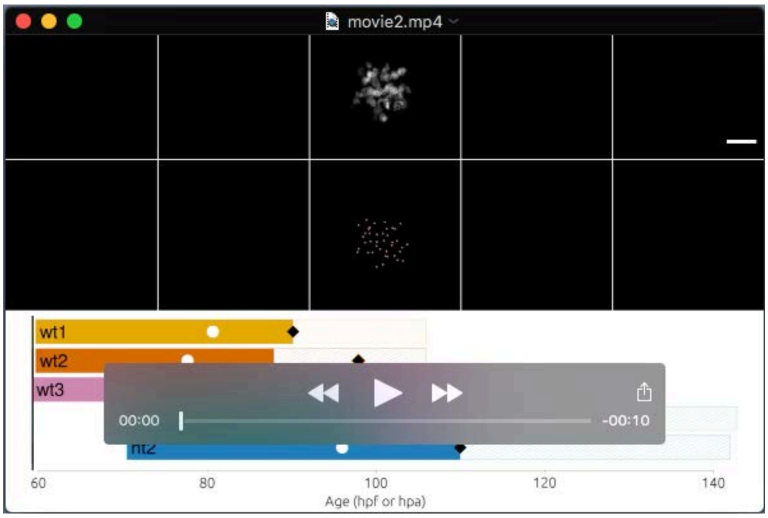

Movie 2. Raw and reconstructed data for five embryos: 3 wild types and 2 clones. Color code as in Fig. 1. (**First row**) Z-projection of 3D volumes. (**Second row**) Z-projection of reconstructed embryos synchronized with the raw data displayed in the first row. Each nucleus approximates a center and is represented by a sphere. Scale bar: 25µm for the first and second rows. (**Third row**) Timeline of the imaging sequences. The cell lineage reconstruction is limited to the colored part. White disc: first collapse. Black diamond: hatching. Age based on fertilization time (wild-type embryos) and activation time (clones).

Development • Supplementary information

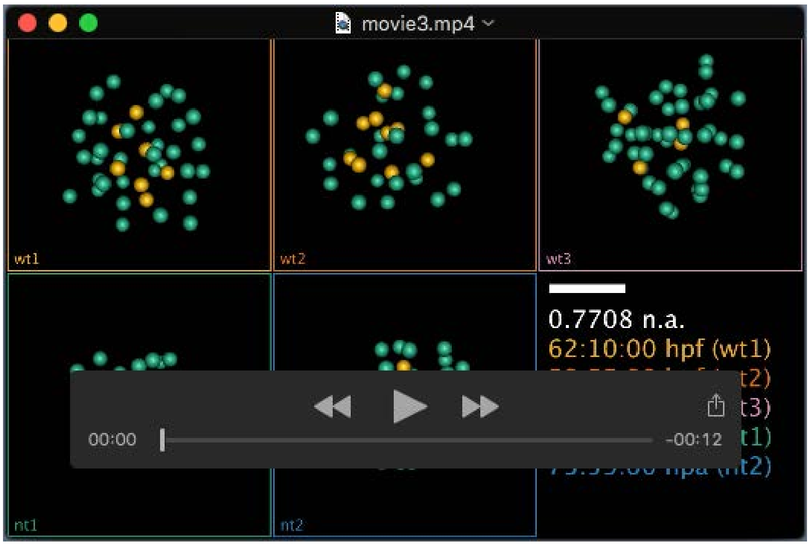

Movie 3. Inner cells and outer cells in temporally rescaled and synchronized digital specimens. Inner cells: yellow spheres. Outer cells: blue-green spheres. Time is given in normalized age (n.a.) and in experimental age for the different embryos with the color code used in Fig. 1. Scale bar: 25 µm.

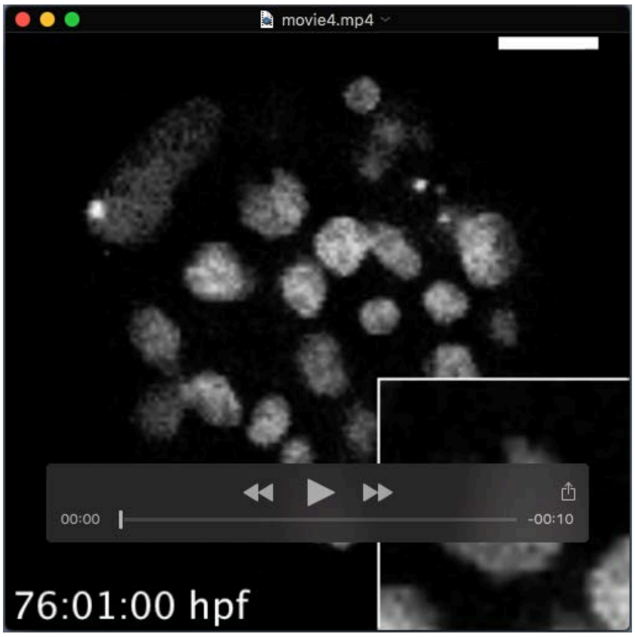

Movie 4. Cell death annotation (in wt3). Z projection of a 10.5 µm thin section (10 slices) of wt3 starting at 76 h.p.f, z-centered around a dying cell. Scale bar: 25 µm. Inset: 2x magnification of the dying cell.

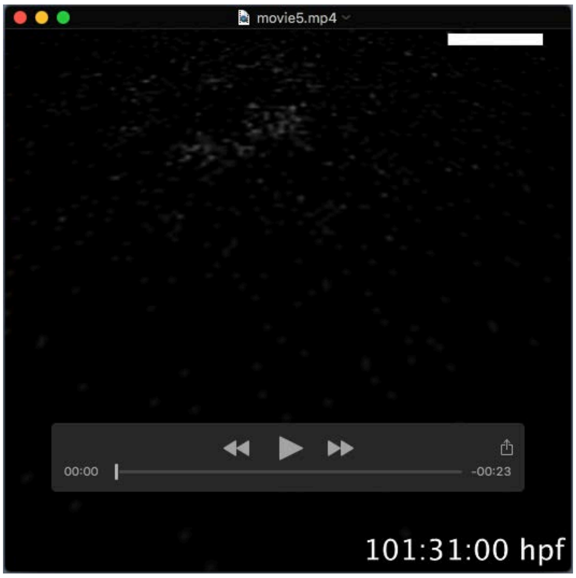

Movie 5. Asymmetric divisions in inner cell population (in wt3). An inner cell (yellow) undergoes an asymmetric division and displays a prophase pattern by 101.5 h.p.f. 15 minutes later, the metaphase plate is parallel to the embryo surface. The cell of interest generates an outer daughter (blue-green, right side of the movie) and an inner daughter (yellow, left side of the movie) by 102 h.p.f. 30 minutes later, the inner nucleus has reached the inner cell mass. Scale bar: 25 µm.

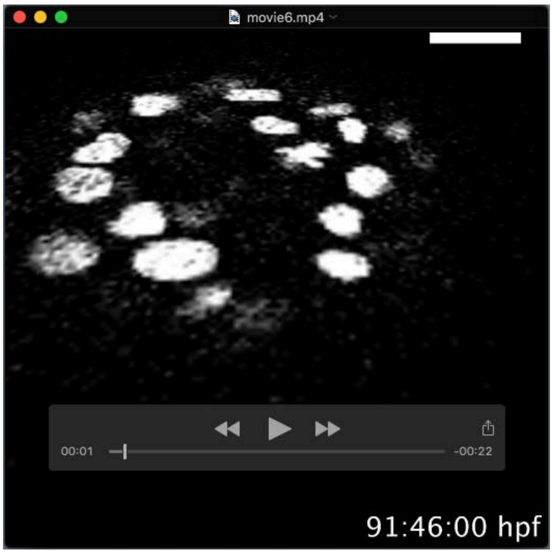

Movie 6. Asymmetric divisions in outer cell population (in wt3). An outer cell (blue-green) undergoes an asymmetric division and displays a prophase pattern by 91.75 h.p.f. 15 minutes later, the metaphase plate is parallel to the imaging plane. The mitotic cell gives rise to an outer daughter (blue-green, right side of the movie) and an inner daughter (yellow, left side of the movie) by 92.25 h.p.f. 60 minutes later, the inner nucleus has reached the inner cell mass. Scale bar: 25 µm.
